# Effect of ultrasonic streaming on intra-dentinal disinfection and penetration of calcium hydroxide paste in endodontic treatment

**DOI:** 10.1590/1678-775720150553

**Published:** 2016

**Authors:** Marcela Paola Castro ARIAS, Amanda Garcia Alves MALIZA, Raquel Zanin MIDENA, Márcia Sirlene Zardin GRAEFF, Marco Antonio Húngaro DUARTE, Flaviana Bombarda de ANDRADE

**Affiliations:** 1 - Universidade de São Paulo, Faculdade de Odontologia de Bauru, Departamento de Dentística, Endodontia e Materiais Odontológicos, Bauru, SP, Brasil.; 2 - Universidade de São Paulo, Faculdade de Odontologia de Bauru, Centro Integrado de Pesquisa, Bauru, SP, Brasil.

**Keywords:** Endodontics, Calcium Hydroxide, Enterococcus faecalis, Ultrasound

## Abstract

**Objective:**

The antimicrobial effect of ultrasonic agitation of calcium hydroxide (CH) pastes in infected bovine dentin and their penetrability were evaluated using confocal laser scanning microscopy (CLSM) and microbiological culture.

**Material and Methods:**

Fifty-two bovine teeth were infected with *Enterococcus faecalis* using a new contamination protocol; then they received CH paste and were divided into groups with or without ultrasound. Ultrasonic agitation was conducted for 1 min with a plain point insert. After 15 d, the CLSM analyzed the viable and dead bacteria with Live and Dead assay. The dentinal wall debris was collected by burs, and the colony forming units (CFU/mL) were counted. The penetrability of the paste inside dentinal tubules was tested using the B-rodamine dye.

**Results:**

The calcium hydroxide paste showed better results with the use of ultrasonic agitation (p<0.05).

**Conclusion:**

The ultrasonic agitation of CH paste increased its antimicrobial action and was responsible for intradentinal penetration with the fulfilment of the tubules.

## INTRODUCTION

Bacterial invasion in the dentin due to caries or fracture results in inflammation, necrosis of the dental pulp, root canal infection, and apical periodontitis^18^. To reduce bacteria during endodontic treatment, instrumentation, antimicrobial irrigants, and intracanal medication should be used^27^. However, some bacteria, such as *Enterococcus faecalis*, are resistant to endodontic treatment, confirmed with a high intratubular penetration^17^ and an adaptive response in the alkaline pH^8^. Therefore, many substances and procedures have been studied to improve the antimicrobial activity of intracanal medications^22^.

Some walls, ramifications, and isthmuses can remain untouched during instrumentation, especially when using mechanized preparation^21^. Anatomical complexities are an important factor to consider in endodontic treatment^29^, and all possible efforts should be made to eliminate bacteria.

Calcium hydroxide paste is an efficient intracanal medication because of its dissociation in calcium and hydroxyl ions^13^, which are responsible for the alkalization of the environment^28^. Its high pH induces hard tissue formation through mineralization and is also responsible for its bactericidal effect, with a pronounced destructive effect on the bacterial cell membranes. However, CH is not cited as a universal intracanal medication since it is not equally effective against all of the microorganisms found in the root canal — such as enterococci, which can tolerate high pH levels^14^ — although it can kill *E. faecalis* in a direct contact^10^.

The real effect of calcium hydroxide pastes over bacteria, especially over *Enterococcus faecalis*, is very controversial. Some *in vitro* research with different methodologies have shown opposite results. Based on direct contact and diffusion agar tests, Gomes, et al.^13^ (2006) observed that 2% CHX gel + Ca(OH)_2_ showed better antimicrobial activity than Ca(OH)_2_ manipulated with sterile water. Evans, et al.^9^ (2003) observed the same result against *E. faecalis* in dentinal tubules. On the other hand, antimicrobial components such as chlorhexidine did not improve the antimicrobial activity of calcium hydroxide against *E. faecalis,* according to Midena, et al.^22^ (2015). Waltimo, et al.^30^ (1999) showed that in a saturated calcium hydroxide solution (pH≥12.5), *in vitro* enterococci were killed within 20 min and yeasts within 6 h. Saturated calcium hydroxide killed *E. faecalis* cells in a few minutes, whereas this time was extended to more than 24 h with the addition of dentin powder^16^. Also, *E. faecalis* was very sensitive in the logarithmic growth phase and was killed by calcium hydroxide in just a few seconds. During the starvation phase, it takes longer^24^. Calcium hydroxide rapidly killed bacteria in *in vitro* conditions; in the root canal it was possible to achieve only partial disinfection of the surface of the canal wall, whereas calcium hydroxide was relatively ineffective in the subsurface layers of the dentin^17^.

*In vivo* studies demonstrated that calcium hydroxide pastes can be effective to decontaminate root canals, depending on the time of maintenance^27^. Gondim, et al.^14^ (2012) showed, by using the quantitative real-time polymerase chain reaction (qRT-PCR) technique, that the association of chlorhexidine with calcium hydroxide did not increase the antibacterial activity of the intracanal medication.

Ultrasound is used for many purposes in endodontics, such as agitation of irrigants, root canal instrumentation, fillings, and removal of medications and posts^23^, but not for agitation of medications.

The aim of this study was to evaluate the antimicrobial activity of calcium hydroxide paste (CH) inside root canals with or without ultrasonic agitation (U). Confocal laser scanning microscopy (CLSM) and microbiological culture techniques were used to detect *Enterococcus faecalis* inside the infected dentinal tubules of bovine origin after chemical action of the paste and physical agitation of the ultrasound. The penetrability of the paste into dentinal tubules with or without ultrasonic agitation was also tested.

## MATERIAL AND METHODS

### Specimen preparation

Fifty-two non-carious bovine teeth acquired from a slaughterhouse were selected and stored in 1% sodium hypochlorite for 48 h. The crown and the apical 5 millimeters of the root were cut off. A root segment with a length of 12 mm was standardized from this apical section in a cutting machine (Isomet, Buehler, IL, USA). Each root canal was enlarged to the size of a #120 K file (Dentsply/Maillefer, Ballaigues, Vaud, Switzerland), and the smear layer was removed using 17% EDTA for 3 min. Removal of the smear layer was confirmed in a previous pilot test in a scanning electron microscope (SEM; Jeol, JSM T220A, Tokyo, Kanto, Japan). The teeth were washed with sterile water for 10 min. The specimens received a double layer of red nail polish (L’Oréal Colorama, Rio de Janeiro, RJ, Brazil) externally to promote posterior contamination only by the main root canal. After complete drying (24 h), the specimens were sterilized by autoclave (Cristofoli, Campo Mourão, PR, Brazil) for 20 min at 121°C. The specimens were incubated in 5 mL of BHI broth (Brain Heart Infusion, Difco, Detroit, MI, USA) at 37°C for 24 h, and CLSM analysis in some specimens confirmed their sterility.

### Dentin contamination

Isolated colonies of *E. faecalis* (ATCC 29212) were used to create the bacterial inocula in BHI suspension after successive cultures. The cell suspension was spectrophotometrically (Bel Photonics, Osasco, SP, Brazil) measured according to the turbidity of the McFarland scale and adjusted to 3x10^8^ CFU/mL. This suspension was incubated for 7 h to achieve the exponential growth of *E. faecalis* based on the growth curve established previously.

The protocol of Andrade, et al.^1^ (2015) was followed to infect the dentin. Under a hood, the specimens were individually inserted into microtubes with 1 mL of sterilized BHI solution and transferred to an ultrasonic bath for 15 min to allow the culture medium to penetrate the dentinal tubules. Bovine teeth are large, so it was not necessary to fix them inside microtubes. The BHI inoculum was added into the microtubes by a pipette (Nichipet EX, Nichiryo, Tokyo, Kanto, Japan) and the centrifugation protocol was performed according to Ma, et al.^20^ (2011). The BHI solution was changed after 24 h, and the centrifugation protocol was repeated after 48 h. All specimens were contaminated after 5 d, when the effectiveness of the contamination protocol was confirmed by the observation at the CLSM.

### Medication

Before insertion of the medication the specimens were divided into 2 groups: Group 1 (n=20) received CH (calcium hydroxide with propylene glycol) paste and Group 2 (n=20) received CH+U (calcium hydroxide with propylene glycol and ultrasonic agitation). The calcium hydroxide paste with propylene glycol (CH) was manipulated in a 3:1 ratio using sterilized materials under a hood.

The specimens were placed on the sterilized stainless steel table device. To remove non-adherent bacteria from the root canal walls, the specimens were irrigated with saline and 17% EDTA solution for 3 min. The root canals were dried with sterilized absorbent paper points, and the medications were inserted with a K 90 manual instrument (Dentsply Maillefer, Ballaigues, Vaud, Switzerland) until the whole extension, checked by the fulfilling at the lower portion of the root canals. After that, the lower apertures were sealed with temporary cement (Coltosol, Vigodent Coltene, Rio de Janeiro, RJ, Brazil).

The teeth of Group 2 received ultrasonic agitation using an ultrasonic device activated by a piezoelectric ceramic pellet system at a frequency of 30,000 Hz (Jet Sonic, Gnatus, Ribeirão Preto, SP, Brazil) by a plain insert (TU13, Trinit Periodontology, São Paulo, SP, Brazil) for 1 min. Ultrasonic agitation was conducted with vertical movements in the buccal-lingual and mesial-distal directions (30 seconds for each direction) in “endo mode” (50% potency).

All of the root canals were sealed with the temporary cement in the upper aperture and incubated for 15 d at 37°C in sterilized microtubes.

Six specimens of positive controls were contaminated but did not receive insertion of the medication. Six negative controls were not contaminated but received insertion of the medication and ultrasonic agitation.

### CLSM analysis

Ten teeth of each group were used in the CLSM analysis. Longitudinal sections of the specimens were created with a diamond disc and saline sterilized solution in an Isomet machine (Isomet, Buehler, IL, EUA) to obtain two halves. Next, 17% EDTA was utilized for 5 min to remove the cut smear layer and then washed in saline solution. The halves of the dentinal tubes were stained with 30 µL of Live and Dead^®^ dye for 10 min.

The samples were examined on an inverted Leica TCS-SPE confocal microscope (Leica Microsystems GmbH, Mannheim, Baden-Württemberg, Germany) using a 40X magnification oil lens. The pictures were obtained by using 23 sections of 1 µm step size in a format of 1024x1024 pixels. Four images were taken of the cervical and middle thirds of each specimen half. For each third, the images were taken in (A) the most superficial region near the canal and (B) in the greater dentinal depth. Images were acquired using the Leica Application Suite-Advanced Fluorescence software (LAS AF, Leica Mannheim, Baden-Württemberg, Germany). The evaluation of bacterial viability was made with the bioImageL TM v21 software^4^ for the bacterial count (green/live and red/dead).

### Microbiological culture analysis

Ten teeth of each group were used in the microbiological culture analysis. After the medications were removed with citric acid, saline solution, and sterilized manual instruments, the specimens were positioned for microbiological sampling. Largo burs (#5 and #6) with low speed (410 rpm) were used for 10 seconds to collect the dentin debris from the internal surface and directly dropped into the microtubes with fresh sterilized BHI solution. These tubes were homogenized and diluted to perform the counting of the CFUs. Then, 50 µL of the content from the microtubes with the dentin debris was spread on Petri dishes and incubated at 37°C for 48 h. All of these steps were accomplished with a sterilized stainless steel table device to avoid hand contact with the specimens.

### Penetrability

Twenty other bovine teeth were prepared in the same manner to evaluate the penetration of Ca(OH)_2_ pastes inside the tubules with or without the use of ultrasound. The teeth were disinfected and cut to remain 12 mm in height. The inner diameters were standardized at 1.2 mm and irrigated with EDTA and saline solution.

The lower portions of the specimens were sealed with Coltosol temporary cement and divided into two medication groups to receive Ca(OH)_2_ paste with agitation by ultrasound for 1 min or with no agitation. The paste was prepared with the addition of 1% rodamin dye to be detected by the CLSM and was inserted into the root canals with a K 90 manual instrument (Dentsply Maillefer, Ballaigues, Vaud, Switzerland) until they were completely full. These specimens were stored at 37°C for 48 h. After that, they were transversally cut every 3 millimeters from the lower portion to obtain three slices of dentin. Sequential images were obtained of each third. The specimens were evaluated and scored by three calibrated examiners as follows: 0= no medication inside dentinal tubules; 1= discrete penetration inside tubules near the root canal space; 2= deep penetration inside tubules, but without fulfilling them; 3= total fulfilment of dentinal tubules.

### Statistical analysis

After normality testing, the non-parametric Kruskal-Wallis test was performed for global comparison, and Dunn test was performed to individual comparisons in the software GraphPad Prism (GraphPad San Diego, CA, USA). A *p* value of <0.05 was considered statistically significant.

## RESULTS

### CLSM analysis

**Figure 1:**
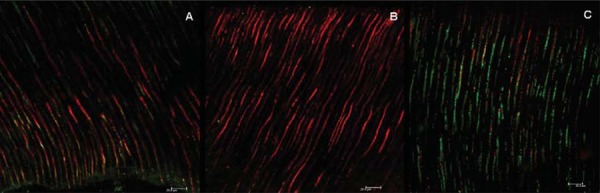
CLSM images after medication for 15 d. A: calcium hydroxide group. B: calcium hydroxide with ultrasonic agitation. C: positive control after contamination protocol without medication. Bars: 20.0 µm. Stained bacteria inside tubules are green or red, which means alive or dead, respectively

**Figure 2:**
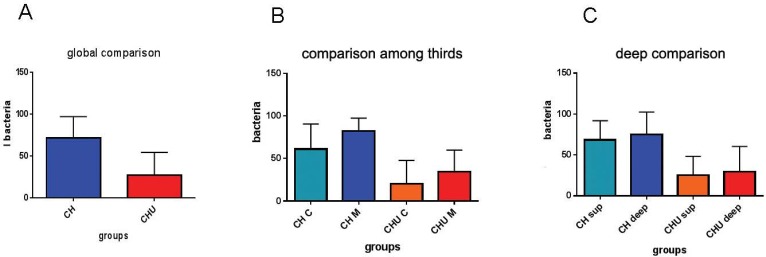
Graphics of median and min-max values of bacterial viability. A: Global comparison of the two groups after intracanal medication, with ultrasonic agitation (CHU) or without (CH). B: Bacterial viability in different thirds, with or without ultrasound, cervical (CHC and CHUC) and medium (CHM and CHUM). C: Bacterial viability in different depths, with or without ultrasound, superficial (CH sup and CHU sup) or deep (CH deep and CHU deep)

Fifteen days after medication insertion, the CLSM results demonstrated a greater amount of dead bacteria for Group 2 (Figure 1). The bacterial viability was higher when only calcium hydroxide paste was used (Group 1- CH) (70%), statistically similar to the positive control group (100%). The use of the ultrasonic device improved the antimicrobial action of the calcium hydroxide (30% viability) (p<0.0001) (Figure 2A).

The cervical and middle thirds were analyzed separately. The results showed that the medication was more effective in the cervical third. There was statistical significance difference between groups with or without ultrasound, as seen at the cervical third, CH C x CHU C – (p=0.0006). Also, the Ca(OH)_2_ without ultrasound at the medium third — CHM — was statistically different from CHU M (p=0.0002) and CHU C (p<0.0001) (Figure 2B).

Two different depths from the root canal into the dentinal mass were analyzed: the area closest to the main root canal (superficial) and the farthest one (deep). In Group 1, the CH in superficial areas had more viable bacteria than the superficial CHU (p=0.0004) and deep CHU (p=0.0033). Group 1’s CH in deep areas had more viable bacteria than superficial CHU (p<0.0001) and deep CHU (p=0.0002) (Figure 2C).

### Microbiologic culture

**Figure 3:**
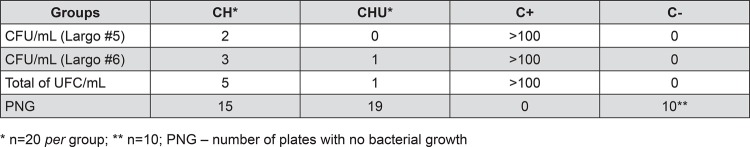
Amount of CFU/mL *per* group after collection with Largo burs #5 and #6; amount of total CFU/mL and amount of plates that did not present bacterial growth

Group 1 presented more CFUs in the specimens than the other group when both Largo burs were utilized (in the superficial and deeper layers of root canal dentin). The group with ultrasonic agitation showed only one colony in a plate of Largo #6 (Figure 3).

The difference occurred between the group medicated with Ca(OH)_2_ with ultrasound and the positive control of bacterial growth (p=0.0002). Group 1 had an intermediate behavior and was not statistically different from Group 2.

### Penetrability

**Figure 4:**
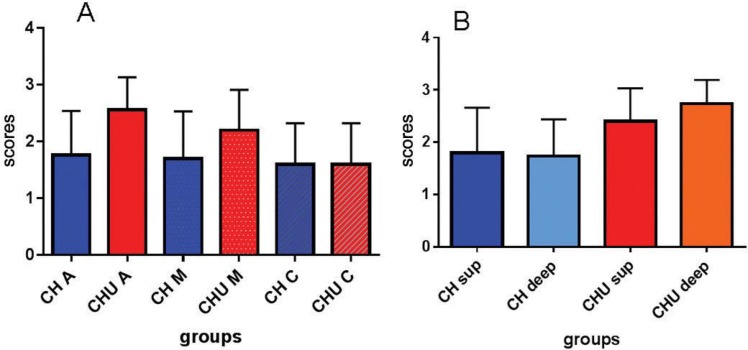
Comparison of scores of intracanal medication penetration inside tubules of the two groups, calcium hydroxide (CH) and calcium hydroxide with ultrasonic agitation (CHU). A: Comparison of penetrability in different thirds of the root canal, apical (CHA, CHUA), medium (CHM, CHUM), and cervical (CHC, CHUC). B: Comparison of penetrability in different depths of dentinal mass, superficial (CH sup and CHU sup) or deep (CH deep and CHU deep)

The transversal slices were read at CLSM, processed and evaluated by the calibrated examiners. Considering the thirds, apical, medium, and cervical, the best penetration occurred at the apical slice with Ca(OH)_2_ and ultrasound (CHUA), which was statistically different from the groups apical Ca(OH)_2_ (CHA) (p=0.0018), medium Ca(OH)_2_ (CHM) (p=0.0004), cervical Ca(OH)_2_ (CHC) (p<0.0001), and cervical Ca(OH)_2_ with ultrasound (CHUC) (p<0.0001) (Figure 4A).

**Figure 5:**
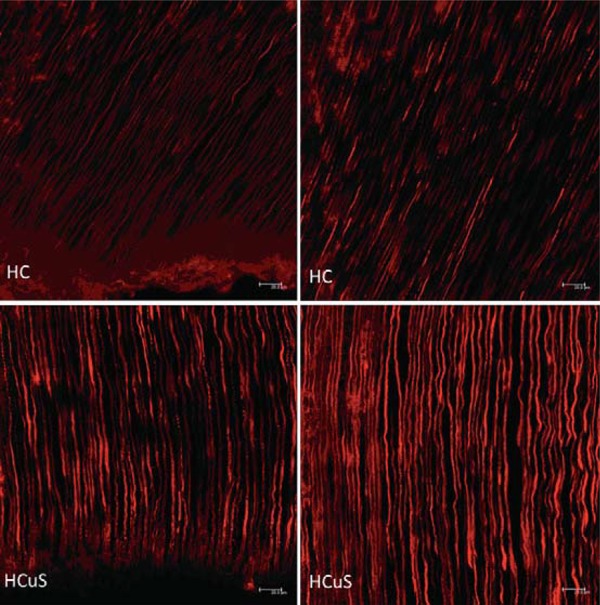
Images by CLSM after penetration of medication stained with 1% rodamin. A and B (HC): calcium hydroxide group. C and D: calcium hydroxide with ultrasonic agitation (HCuS). Bars: 20.0 µm

When the slices were analyzed regarding the depth areas of the specimens, the group that received Ca(OH)_2_ with ultrasound (CHU) showed fulfilling of the dentinal tubules in both superficial and deep areas. The group CHU deep was statistically different from groups without ultrasound, i.e., the Ca(OH)_2_ at the superficial area (p=0.008) and the Ca(OH)_2_ at the deep area (p=0.0026) (Figure 4B and 5).

## DISCUSSION

Bacteria’s ability to penetrate into the dentin can be determined through microbiological analyses from the number of colony-forming units^17^, the number of infected tubules in the histological sample^19^, or the presence of bacteria in the root canal walls^2^. Each one of these methods has its advantages.

Confocal laser scanning microscopy (CLSM) permits the obtainment of a series of optical sections as thin as 0.3 µm of intact biological samples. It is commonly used with vital staining techniques to determine the viability profile, architecture, and spatial distribution in microbial biofilms^6^ ^,^ ^31^. Moreover, it is possible to observe the infection of dentinal tubules and to differentiate live and dead bacteria.

Previous studies^15^ have shown that CLSM is able to detect the viability of bacteria in specimens of carious lesions via the use of the immunofluorescence technique and can provide information about the intensity of the dentinal infection within the dentinal tubules^11^. In this work, the criteria to establish an adequate methodology consisted of determining the bacterial viability by CLSM. To achieve this, the experimental model tried to simulate the real situation using bovine dentinal blocks, which are similar to human dentin in structure^3^.

Two different methodologies of analysis were utilized in this study to verify the antimicrobial action of medication pastes inside bovine root canal: MC and CLSM. The similar results of the CLSM and the microbiological culture proved that these methodologies can be safely used for this type of experiment.

The bovine dentinal block model and the *Enterococcus faecalis* bacterial strain have been commonly used as a reference to test endodontic medications^9^ ^,^ ^25^ since the Haapasalo and Ørstavik study^17^ (1987). The *E. faecalis* strain has shown an ability to infect the dentinal tubules^17^ to tolerate high pH levels^5^ and to survive in sealed root canals^26^. It is also the most prevalent bacterial strain in endodontic cases with persistent lesions^12^.

The bovine dentin contamination established in the Ma, et al.^20^ (2011) study suffered some modifications. An ultrasonic bath, a new inocula period based on the *E. faecalis* growth curve and number of days of bacterial growth were added to the new dentin contamination protocol. Therefore, it was possible to have a homogeneous contamination and high bacterial viability rates within the dentinal tubules^1^.

Slicing the dentinal tubes into two halves was preferable to fracturing because some fractured fragments did not provide satisfactory CLSM images. Furthermore, the use of a diamond disk for slicing was necessary to obtain a regular and plain surface, which improved the focus adjustment. After slicing, the smear layer was removed with 17% EDTA for 10 min, and the image of microorganisms within the tubules could then be captured. EDTA has a capacity to kill microorganisms but is a weak acid^32^. With proper application time, EDTA could remove the smear layer without elimination of microorganisms, which is why only EDTA was used. Therefore, the contamination of the specimens was higher and deeper than in the tests that confirmed that EDTA presents antimicrobial capacity. A previous study^1^ verified that the influence of this superficial EDTA application over the bacterial viability was not observed.

The results showed that calcium hydroxide presented less antimicrobial action against *E. faecalis* when used only with propylene glycol (Group 1). However, combining it with mechanical agitation with an ultrasonic device (Group 2) resulted in lower bacterial viability. This information, according to Duarte, et al.^7^ (2012), demonstrated that the ultrasonic agitation of CH pastes favored a higher pH level and calcium ion release in simulated external root resorptions. The hypothesis is that the results probably occurred because of the higher ionization that such an apparatus can provide or because of the higher penetration of the CH pastes into the dentinal tubules. Also, the lesser surface tension of propylene glycol allowed for greater ion penetration.

According to Haapasalo and Orstavik^17^ (1987) a calcium hydroxide paste called Calasept failed to eliminate *E. faecalis* from tubules, even superficially, a result confirmed in Evans, et al.’s ^9^ (2003) study. Some researchers have attested that calcium hydroxide is ineffective against *E. faecalis*; however, others disagree because of the methodological differences^10^. *In vivo* researches have shown better results for calcium hydroxide^14^ ^,^ ^27^. Thus, we are proposing an easy and good way to suppress the limitations of calcium hydroxide paste to eliminate bacteria hidden deep inside tubules.

This study has shown that the viability of bacteria in the infected dentin can be effectively determined *in vitro* by CLSM and MC. The use of ultrasonic agitation for one minute on the intracanal medication is a simple way to achieve better antimicrobial results for the CH pastes, improving results in the cervical and middle thirds, especially when analyzing the deepest portion of the dentinal tubules.

## CONCLUSION

The partial dentinal disinfection can be achieved by calcium hydroxide paste, but its performance is improved by ultrasonic agitation (physical effect) of the pastes. Ultrasonic agitation of the intracanal medication promoted greater and deeper penetration and antimicrobial action inside the dentinal tubules.
